# How Many HIV Infections May Be Averted by Targeting Primary Infection in Men Who Have Sex With Men? Quantification of Changes in Transmission-Risk Behavior, Using an Individual-Based Model

**DOI:** 10.1093/infdis/jiu470

**Published:** 2014-12-01

**Authors:** Peter J. White, Julie Fox, Jonathan Weber, Sarah Fidler, Helen Ward

**Affiliations:** 1 MRC Centre for Outbreak Analysis and Modelling; 2 NIHR Health Protection Research Unit in Modelling Methodology; 3 Department of Infectious Disease Epidemiology; 4 Department of Genitourinary Medicine and Infectious Disease, Faculty of Medicine, Imperial College London; 5 Modelling and Economics Unit, Public Health England Centre for Infectious Disease Surveillance and Control; 6 Department of HIV, Faculty of Medicine, Guys and St Thomas' NHS Trust / Kings College London, United Kingdom

**Keywords:** men who have sex with men, primary HIV infection, diagnosis, counseling, behavior change, HIV transmission risk, infections averted, mathematical model

## Abstract

In the United Kingdom, human immunodeficiency virus (HIV) transmission among men who have sex with men (MSM) is not under control, despite readily available treatment, highlighting the need to design a cost-effective combination prevention package. MSM report significantly reduced transmission risk behavior following HIV diagnosis. To assess the effectiveness of HIV diagnosis in averting transmission during highly infectious primary HIV infection (PHI), we developed a stochastic individual-based model to calculate the number of HIV-transmission events expected to occur from a cohort of recently infected MSM with and those without the behavior changes reported after diagnosis. The model incorporates different types of sex acts, incorporates condom use, and distinguishes between regular and casual sex partners. The impact on transmission in the 3 months after infection depends on PHI duration and testing frequency. If PHI lasts for 3 months and testing is performed monthly, then behavior changes after diagnosis would have reduced estimated transmission events by 49%–52%, from 31–45 to 15–23 events; a shorter duration of PHI and/or a lower testing frequency reduces the number of infections averted. Diagnosing HIV during PHI can markedly reduce transmission by changing transmission-risk behavior. Because of the high infectivity but short duration of PHI, even short-term behavior change can significantly reduce transmission. Our quantification of the number of infections averted is an essential component of assessment of the cost-effectiveness of strategies to increase detection and diagnoses of PHI.

In the United Kingdom, transmission of human immunodeficiency virus (HIV) among men who have sex with men (MSM) has continued to remain high, despite the ready availability of treatment [1–3]. Despite the high efficacy of treatment as prevention (TasP) [4], Brown et al [5] report that TasP will have little impact in the United Kingdom, where treatment is readily available to those with diagnosed HIV infection and there is relatively little transmission from those with diagnosed infection who are not receiving treatment. There is a need for more-widespread and more-frequent testing to identify undiagnosed infections [3, 5] and for more-effective interventions [3] as part of a combination of measures.

Primary HIV infection (PHI) is highly infectious (because of the associated high viral load) and is often associated with high-risk sexual behavior [6]. However, it is also transient, and its importance in HIV transmission is debated and depends upon context [7]. A recent modeling study estimated that 48% of HIV infections acquired by MSM in the United Kingdom arose from undiagnosed PHI [3], which is in line with phylogenetic studies [8, 9]. Therefore, diagnosing HIV infection in high-risk individuals during PHI may be highly effective if it facilitates effective reduction of the transmission risk. However, diagnosing HIV infection during PHI requires frequent testing, which is likely to be expensive and difficult to achieve, so it is important to quantify the benefits of increased efforts to diagnose PHI infection.

We previously reported that most high-risk MSM in whom HIV infection was recently diagnosed and who underwent standard safer-sex counseling during care changed their sexual behavior [10]. In general, they reported an increased frequency of condom use and fewer sex partners, thereby reducing their risk of transmission, for at least the 3-month follow-up period studied [10]. To assess the effectiveness of early diagnosis of HIV infection in high-risk MSM to prevent HIV transmission, we developed a stochastic, individual-based mathematical model to quantify the number of HIV transmission events expected to be averted by the postdiagnosis behavior change, accounting for increased infectivity during PHI and uncertainty in parameter estimates, including the duration of PHI.

## METHODS

**Figure 1.**
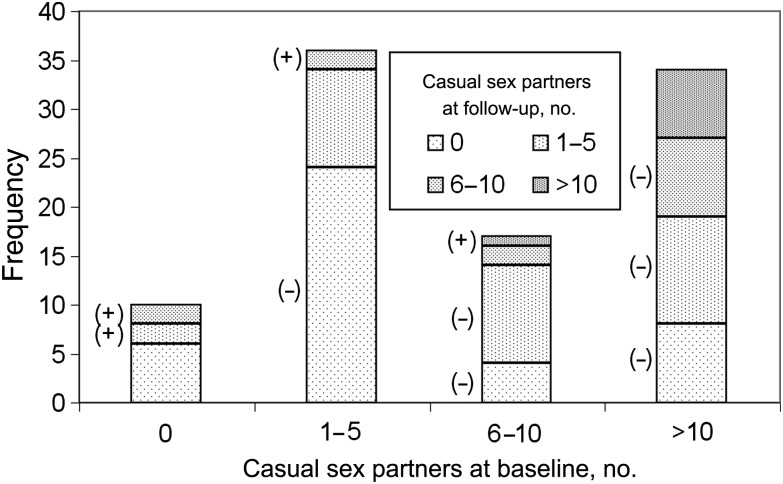
Reported numbers of casual sex partners in the 3-month periods before and after diagnosis of human immunodeficiency virus infection. The symbol (+) represents an increase in the number of casual partners after diagnosis, the symbol (−) represents a decrease, and the absence of a symbol represents no change. Most respondents greatly reduced their numbers of casual partners, with many reporting none, although a few reported increased numbers. Data are from Fox et al [9].

A mathematical model, described below, was developed to analyze the results of the study in London, United Kingdom, by Fox et al [10], who interviewed MSM with recently acquired HIV infection about their sexual behavior in the 3-month periods before and after HIV diagnosis, using a standardized questionnaire. At baseline (median, 7 days after diagnosis) and 3-month follow-up, participants reported their number of sex partners (regular and casual) in the previous 3 months, the frequency of types of sex acts (insertive anal, receptive anal, insertive oral and receptive oral), and the frequency of condom use (always, sometimes, or never) for each type of sex act with each type of partner, and the HIV status of their regular partner (positive, negative, unknown, or not applicable); the HIV status of casual partners was not reported [10]. Ninety-eight of 104 eligible MSM were enrolled, and there was 100% follow-up. Participants were offered antiretroviral therapy (ART) at diagnosis, which 73 accepted; there was no difference in the behavior change in those who did or did not accept ART (χ^2^ = 0.224; *P* = .636). In this analysis we are interested in the effects of behavior change and do not consider the effects of ART on transmission from those who received it. Most participants (96%) were white; the median age was 33 years (range, 20–59 years). At baseline, 52 reported a regular partner, with 10 reporting that they were monogamous. Of the 52 regular partners, 24 were HIV negative and 18 were HIV positive at the time of diagnosis of the index case, and 10 had an unknown HIV status. The numbers of casual sex partners and changes after diagnosis are illustrated in Figure 1.

A stochastic, individual-based Monte Carlo simulation was developed to calculate the number of transmission events that would have occurred from each study participant during the 3-month period after infection under the following scenarios: (1) HIV infection had not been diagnosed, and therefore the prediagnosis behavior pattern had occurred throughout the 3 months; (2) HIV infection was diagnosed immediately, so that the postdiagnosis behavior pattern had occurred throughout the 3 months; and (3) since immediate diagnosis of HIV will be rare, the effects of different testing frequencies, ranging from twice per month to once every 2 months, was also examined, so the proportion of PHI during which the postdiagnosis behavior change occurs varies.

For each scenario examined, 10 000 model realizations were performed.

In each model realization, the number of partners of each respondent who became infected was determined randomly, by first determining randomly how many of the partners were HIV negative (and therefore at risk of becoming infected) and then determining randomly which of those partners became infected, based on the probability of transmission, which was calculated using a Bernoulli-process model. This was based on the risk score model of Fox et al [11], with the important difference that the risk score calculates the risk of an HIV-negative individual acquiring HIV, which can only happen once, whereas our model calculates the expected number of HIV transmission events from an HIV-positive individual.

The probability of transmission occurring from an HIV-positive individual to an HIV-negative partner is

$$1-\underset{i}{\Pi }{\left[\text{1-}\sigma \beta_{\text{i}}\text{(1-}\sigma_{i}\text{)}\right]}^{{\text{N}}_{\text{i,j}}^{\text{C}}}{\left(1-\sigma \beta_{\text{i}}\right)}^{N_{i,j}^{N}}$$

The expected number of transmission events from an HIV-positive individual, considering all of this person's partners and the proportion of those partners who are not already HIV infected, is

$$\sum_{j}N_{j}(1-\pi_{j})\left\{1-{\prod_{i}\left[1-\varphi \beta_{i}(1-\sigma_{i})\right]}^{N_{i,j}^{C}}{\left(1-\varphi \beta_{i}\right)}^{N_{i,j}^{N}}\right\},$$

where *i* is the sex-act type (insertive anal, receptive anal, or insertive oral), *j* is the partner type (regular or casual), *β_i_* is the transmission probability per sex act of type *i*, *φ* is the factor increasing infectivity due to PHI (which takes the value 1 when PHI has ended), *σ_i_* is the proportionate reduction in *β_i_* due to condom use, *π_j_* is the proportion of partners of type *j* who are already infected with HIV (ie, prevalence), *N^C^_i,j_* is the number of sex acts of type *i* with a partner of type *j* in which a condom was used, *N^N^_i,j_* is the number of sex acts of type *i* with a partner of type *j* in which a condom was not used, and *N_j_* is the number of partners of type *j*.

In the model, sex acts of each type were distributed over the partners, because Fox et al [10] asked respondents to specify the frequency of sex acts of each type and the number of partners, rather than to provide a detailed report about their sexual behavior with each individual partner. Because the HIV status of casual partners was not known, it was determined randomly, with the probability of each partner being HIV positive equal to the prevalence of HIV infection among high-risk MSM in London (12.3%; 95% confidence interval [CI], 10.7%–14.1%) [12]. When the HIV status of the regular partner was unknown, it was determined randomly with the probability that the regular partner was HIV positive being the equal to the proportion of regular partners of known HIV status who were HIV-positive (ie, 18 of 42 [43%; 95% CI, 28%–59%]) [10]. If condom use was described as “sometimes,” then condoms were assumed to have been used on 50% of occasions. Condoms were assumed to reduce the per-act transmission probability by 80% (95% CI, 53%–92%) [13].

Transmission probabilities per act for each type of act were taken from literature. For anal sex, 2 recent articles reported different estimates: Scott et al [14] estimated transmission probabilities of 0.73% (95% CI, .45%–.98%) and 0.22% (95% CI, .05%–.39%) when the HIV-positive partner is insertive and receptive, respectively. Corresponding estimates from Patel et al [15] were 1.38% (95% CI, 1.02%–1.86%) and 0.11% (95% CI, .04%–.28%). Note that these are probabilities of transmission from the HIV-positive person*,* who were the respondents in Fox et al's behavioral survey, not probabilities of acquisition by the HIV-negative person, so the probabilities are higher where the respondent is the insertive partner. We compared scenarios using the estimates from Scott et al to scenarios using the estimates from Patel et al for anal sex (the estimates from Vittinghoff et al [16] were intermediate, relative to those from Scott et al and Patel et al). In all cases, the transmission probability used for insertive oral sex was 0.04% (95% CI, .01%–.17%) [16]; receptive oral was assumed to have negligible risk.

Because the duration of PHI is uncertain, we varied the assumed value, using 1.25 months (Hollingsworth et al [17] estimated a lower bound of 1.23 months), 2 months, 2.5 months (the duration used by Wawer et al [18]), and 3 months (the duration for which we have behavioral data), which is the commonly assumed duration [6]. The estimated increase in infectivity of 7.25 (95% CI, 3.05–17.3) [15, 18] is based on an assumed 2.5-month duration of PHI; in the scenarios where the duration of PHI was varied, the infectivity multiplier was adjusted inversely: for example, if PHI lasts 3 months, then it is adjusted by a factor of 2.5/3 = 0.833, whereas if PHI lasts 1.25 months, then it is adjusted by a factor of 2.5/1.25 = 2.

## RESULTS

**Figure 2.**
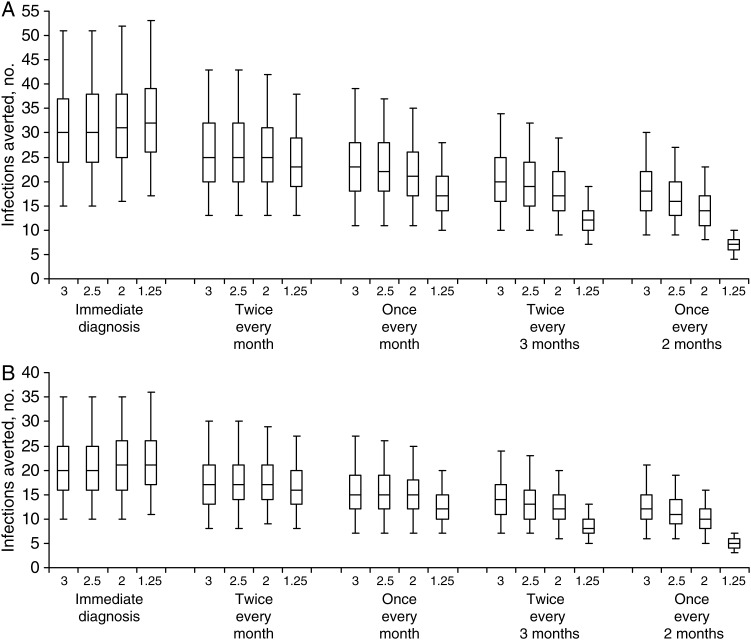
Numbers of infections averted in the 3 months following human immunodeficiency virus (HIV) infection under different scenarios. The graph shows the median, interquartile range, and 95% range of the estimated number of infections averted with different frequencies of testing, using transmission probabilities for anal sex estimated by Patel et al [15] (*A*) and Scott et al [14] (*B*). Numerical labels on the horizontal axis indicate the assumed duration of primary HIV infection (PHI) in months. As the frequency of testing declines, so does the number of infections averted. If the duration of PHI is as short as 1.25 months, then very frequent testing—twice every month—is necessary to avert a substantial number of infections.

In the baseline scenario, in which we examined what would have happened if the modeled MSM had not received a diagnosis of HIV infection and their pattern of behavior reported in the prediagnosis period had continued unchanged throughout the 3 months after infection, then the predicted number of HIV transmission events occurring from the cohort of 98 MSM would have been 31 (95% CI, 14–55) and 33 (95% CI, 17–56), using the parameter values from Scott et al and assuming PHI lasts 3 months or 1.25 months, respectively; corresponding estimates using the values from Patel et al were 45 (95% CI, 22–80) and 48 (95% CI, 25–82), respectively. In the scenario in which all of the modeled MSM received a diagnosis of HIV infection immediately and their postdiagnosis behavior pattern had occurred throughout the 3 months after infection, there would have been a reduction of 65% (using the estimates from Scott et al) or 64% (using the estimates from Patel et al) in the number of transmission events (Figure 2), compared with the respective baseline scenarios. Since immediate diagnosis could not be practicably achieved, we examined the effect of testing twice per month, once per month, twice every 3 months, and once every 2 months (Figure 2). An important cause of uncertainty in the numbers of infections averted is uncertainty in the transmission probabilities associated with anal sex, with the estimates of Patel et al leading to a greater number of infections occurring and therefore a greater number averted by postdiagnosis behavior change. As the frequency of testing declines, so does the number of infections averted. If PHI lasts for 3 months, then testing every month could avert a substantial number of infections. Importantly, if the duration of PHI is as short as 1.25 months, then it is necessary to have very frequent testing—twice per month—to avert a substantial number of infections; otherwise, infection will be undiagnosed for the majority of the period of PHI. Note that our analysis considers the 3-month period after HIV infection, and therefore if diagnosis occurs toward the end of this period then there is little impact in terms of calculated numbers of infections averted; however, the actual number of infections averted would be greater than this because the behavior change associated with the 3-month period after diagnosis [10] would extend beyond the period considered in our analysis.

Importantly, these calculations account for the heterogeneity in the reported behavior change [10]: 74 of 98 MSM in whom HIV was recently diagnosed (76%) changed their behavior to greatly reduce the risk of transmission to others in the 3 months following their HIV diagnosis. Therefore, the postdiagnosis transmission risk in the cohort was concentrated in approximately a quarter of individuals, some of whom increased their numbers of casual partners. However, it is important to note that we do not know whether they were serosorting to reduce their transmission risk or using condoms selectively with partners known to be HIV negative or of unknown status. Therefore, it is possible that we may have overestimated the transmission risk from these individuals.

## DISCUSSION

The incidence of HIV infection among MSM in the United Kingdom has remained high despite the ready availability of ART [1–3], and further intervention efforts are required [3, 5]. Earlier diagnosis of HIV through more-frequent testing is required as part of a package of interventions [5]. However, with limited resources, interventions need to be cost-effective. In this analysis, we assess the effectiveness, in terms of numbers of transmission events averted, of diagnosis of HIV infection in the first 3 months of infection, considering the effect of HIV diagnosis on sexual risk behavior.

Fox et al [10] found that MSM with a recent diagnosis of HIV infection substantially changed their sexual behavior, thereby reducing the risk of onward transmission. The modeling analysis in this article indicates that a significant number of HIV transmission events would be averted by this behavior change if it were to occur during PHI, provided that testing were sufficiently-frequent to detect PHI, which of course depends on the duration of PHI. This quantification of the outcome of interest—the number of infections averted—is an essential component in assessing the cost-effectiveness of interventions where the study end point was behavior change, rather than measured incidence of infection.

Our findings indicate that a policy of frequent testing of those at high risk of HIV infection, to ensure that the decrease in risk behavior, even if only for a limited interval around their most infectious period, has the potential to avert a significant number of infections at the population level, provided that the duration of PHI is not too short for testing at the necessary frequency to be feasible. However, assessment of the cost-effectiveness of an intervention is complex and context specific—it requires consideration of multiple contextual factors, including targeting and configuration of services, the associated costs and uptake of testing by relevant client groups (which determines the average number of tests needed to identify a case of PHI), and the cost-savings and quality-of-life gains that accrue from averting infections—and is therefore beyond the scope of this article. However, more-frequent testing in genitorurinary medicine and HIV services is only one approach to increasing diagnoses of PHI, and enhanced recognition of the symptoms of seroconversion in other healthcare settings contributes to the new proposed self-testing initiatives [19]. Alternatively, rather than routine frequent testing, it might be more cost-effective to encourage testing soon after episodes of high-risk behavior, provided there is clear, well-understood guidance on a what constitutes such behavior.

With around half of new HIV infections arising from individuals with PHI [3, 8], rapid contact tracing involving individuals who recently received a diagnosis of HIV infection could be an effective way to identify PHI cases: even if the initial primary case has progressed from the PHI stage, cosecondary cases might be identified while still in PHI. However, most of the transmission risk reported by the MSM studied by Fox et al [10] involves casual partners, for whom partner notification might not be possible.

Although a significant number of transmission events were calculated to be averted by the behavior change associated with HIV diagnosis and entry into standard care, transmission risk remains, and there is scope for further improvement. ART is likely to be beneficial in reducing transmission from PHI by reducing viral load, and, encouragingly, Fox et al [10] found that choosing ART was not associated with behavioral disinhibition. However, the time taken for viral load to be suppressed [20] will limit ART's impact on transmission from individuals during PHI, and therefore behavior change during this period is vital. As behavior-change interventions are costly and their effects typically transient [21, 22], resources need to be focused where they will be most effective. Fortunately, diagnosis of HIV infection coupled with standard safer-sex counseling was sufficient for three quarters of individuals to eliminate their risk of onward transmission for at least the same length of time as the duration of PHI—they did not require intensive behavior-change interventions [10]. This means that intervention efforts could be targeted at the remaining quarter of patients who did not eliminate their transmission risk, with a small proportion having increased their risk (although we do not know the extent to which it was mitigated by serosorting and/or increased condom use, which were not evaluated in that study).

Strengths of the study upon which this analysis is based are the high participation rate (98 of 104 MSM), the 100% follow-up rate, and the fact that the intervention was standard treatment and counseling and, therefore, was representative of what occurs in practice [10]. However, for casual partners we do not know how numbers of each type of sex act and patterns of condom use were distributed; having this information would have increased the precision of our analysis. Additionally, there are a number of limitations of our analysis, which will result in an underestimation of the benefits of early HIV diagnosis in averting transmission. First, this analysis only considers averted transmission from the index cases and does not consider the additional infections that would otherwise have occurred from the secondary cases that were averted. The longer-term population-level impact of rapid diagnosis of HIV infection depends on factors that include the phase of the epidemic, coverage of the testing, and the presence of other interventions. Second, after HIV diagnosis, reductions in transmission-risk behavior may persist in the longer term beyond the period studied by Fox et al [10]. Third, early HIV diagnosis provides entry into care, facilitating provision of ART and reducing the infectivity of the individual through reductions in viral load. Finally, serosorting behavior—specifically choosing partners known to be HIV positive, for unprotected anal intercourse, to eliminate the risk of transmission to them [23–25]—was not investigated by the empirical study [10], so we do not know the extent to which it occurred, although its occurrence was likely [26]. Serosorting could have significantly further reduced the number of transmission events below the calculations of this article: if casual partners were selected to be HIV positive, then the risk of transmission from study respondents would have been eliminated. (Of course, serosorting is only protective if the serostatus is known accurately; otherwise, the transmission risk is increased [27]). It is possible that inconsistent condom use with casual partners represents consistent condom use with partners who are known to be HIV negative or have an unknown HIV infection status and no condom use with partners known to be HIV positive. Therefore, it would be beneficial if future empirical studies assessed, in more detail, patterns of condom use and serosorting behavior, and assessed behavior over the longer term.

Nevertheless, targeting prevention efforts at high-risk individuals during the period of PHI, so that the marked behavior change associated with recent diagnosis of HIV infection coincides with the period of peak infectivity, possibly supplemented by ART, could be an efficient use of limited HIV-prevention resources.
